# Exploring Deep Transfer Learning Techniques for Alzheimer’s Dementia Detection

**DOI:** 10.3389/fcomp.2021.624683

**Published:** 2021-05-12

**Authors:** Youxiang Zhu, Xiaohui Liang, John A. Batsis, Robert M. Roth

**Affiliations:** 1 Computer Science, University of Massachusetts Boston, Boston, MA, USA; 2 School of Medicine, University of North Carolina, Chapel Hill, NC, USA; 3 Geisel School of Medicine at Dartmouth, Lebanon, NH, USA

**Keywords:** Alzheimer’s Disease, Dementia, Early Detection, Speech analysis, Spontaneous speech, Deep learning, Transfer learning

## Abstract

Examination of speech datasets for detecting dementia, collected via various speech tasks, has revealed links between speech and cognitive abilities. However, the speech dataset available for this research is extremely limited because the collection process of speech and baseline data from patients with dementia in clinical settings is expensive. In this paper, we study the spontaneous speech dataset from a recent ADReSS challenge, a Cookie Theft Picture (CTP) dataset with balanced groups of participants in age, gender, and cognitive status. We explore state-of-the-art deep transfer learning techniques from image, audio, speech, and language domains. We envision that one advantage of transfer learning is to eliminate the design of handcrafted features based on the tasks and datasets. Transfer learning further mitigates the limited dementia-relevant speech data problem by inheriting knowledge from similar but much larger datasets. Specifically, we built a variety of transfer learning models using commonly employed MobileNet (image), YAMNet (audio), Mockingjay (speech), and BERT (text) models. Results indicated that the transfer learning models of text data showed significantly better performance than those of audio data. Performance gains of the text models may be due to the high similarity between the pre-training text dataset and the CTP text dataset. Our multi-modal transfer learning introduced a slight improvement in accuracy, demonstrating that audio and text data provide limited complementary information. Multi-task transfer learning resulted in limited improvements in classification and a negative impact in regression. By analyzing the meaning behind the AD/non-AD labels and Mini-Mental State Examination (MMSE) scores, we observed that the inconsistency between labels and scores could limit the performance of the multi-task learning, especially when the outputs of the single-task models are highly consistent with the corresponding labels/scores. In sum, we conducted a large comparative analysis of varying transfer learning models focusing less on model customization but more on pre-trained models and pre-training datasets. We revealed insightful relations among models, data types, and data labels in this research area.

## 1 INTRODUCTION

The number of patients with Alzheimer’s Disease (AD) over the age of 65 is expected to reach 13.8 million by 2050, leading to a huge demand on the public health system (Association, 2020). While there is no proven effective treatment on AD, considerable effort has been put forth into early detection of AD, such that interventions can be implemented at that stage. Screening measures, neuropsychological assessments, and neuroimaging scans are not pragmatic, cost- or time-efficient approaches for widespread use.

Expressive language impairment is common in AD, such as reduced verbal fluency and syntactic complexity, increased semantic and lexical errors, generating more high-frequency words and shorter utterances, and abnormalities in semantic content (Boschi et al., 2017; Fraser et al., 2016; Mueller et al., 2018a; Sajjadi et al., 2012). Expressive language impairment has also been observed in patients with Mild Cognitive Impairment (MCI), a population at high risk for the development of AD (Kim et al., 2019; Mueller et al., 2018b; Themistocleous et al., 2020). Furthermore, recent meta-analytic and systematic reviews have found that measures of expressive language contribute to the prediction of progression from MCI to AD (Belleville et al., 2017; Prado et al., 2019).

Researchers have explored spontaneous speech as a means of practical and low-cost early detection of dementia symptoms. Pitt Corpus (Becker et al., 1994), one of the large speech datasets, includes spontaneous speech obtained from a Cookie Theft Picture (CTP) description task. Since then, the CTP task has become popular in dementia research and it has been further explored with computerized agents to automate and mobilize the speech collection process (Mirheidari et al., 2017, 2019b) and in other languages including Mandarin (Wang et al., 2019a; Chien et al., 2019), German (Sattler et al., 2015), and Swedish (Fraser et al., 2019b). Other spontaneous speech datasets for dementia research include those collected from film-recall tasks (Tóth et al., 2018), story-retelling tasks (Fraser et al., 2013), map-based tasks (de la Fuente Garcia et al., 2019), and human conversations (Mirheidari et al., 2019a). While a number of studies have investigated speech and language features and machine learning techniques for the detection of AD and MCI, this research field still lacks balanced and standardized datasets on which these different approaches can be systematically and fairly evaluated.

Speech datasets available for dementia research are often small. As shown in Table 1, if we consider AD and non-AD as two classes, the numbers of user-samples in each class are in the hundreds. In the past few years, researchers have explored handcrafted features and machine learning algorithms with these datasets for building classification and regression models. Mueller et al. (2018a) published a survey to show effective linguistic features including semantic content, syntax and morphology, pragmatic language, discourse fluency, speech rate, and speech monitoring. The linguistic features were often identified manually, and the analysis methods were complex and highly task- and data-dependent. Croisile et al. (1996) manually extracted 23 information units from the picture using language knowledge that were effective in dementia detection. Fraser et al. (2019a) developed an auto-generation process of information units for the analysis. Yancheva and Rudzicz (2016) and Fraser et al. (2019b) further proposed to auto-generate topic models that can recall 97% of the human-annotated information units. Similarly, the acoustic-based analysis was started with pre-defined features and recently automated with computational models. Hoffmann et al. (2010) considered acoustic features for each utterance. Fraser et al. (2013) evaluated the statistical significance of pause and word acoustic features. Tóth et al. (2015) considered four descriptors for silent/filled pauses and phonemes. Gosztolya et al. (2016) and Tóth et al. (2018) implemented a customized automatic speech recognition (ASR) and automatic feature selection for phones, boundaries, and filled pauses. Haider et al. (2019); Luz et al. (2020) proposed an automatic acoustic analysis approach using the paralinguistic acoustic features of audio segments. However, the performance results of handcrafted features and customized machine learning algorithms are highly dependent on the tasks and datasets. In 2020, the Alzheimer’s Dementia Recognition through Spontaneous Speech (ADReSS) Challenge became the first shared-task event focused on AD detection (Luz et al., 2020). The ADReSS organizers pre-processed the CTP dataset of the Pitt Corpus and provided the same dataset to the challenge participants, enabling a fair competition. The techniques and results in this paper will strictly follow the guideline of the ADReSS Challenge.

In recent years, **transfer learning techniques** have significantly advanced the research on Image Recognition (IR), Automatic Speech Recognition (ASR), and Natural Language Processing (NLP). Transfer learning focuses on storing knowledge gained from an easy-to-obtain large-sized dataset from a general task and applying the knowledge to a downstream task where the downstream data is limited. A typical transfer learning model incorporates a pre-trained model as its backbone and is later customized for the downstream task. The pre-training process is computationally-intensive and requires a dataset of sufficient size. Different pre-trained models result in different performances as they inherit different knowledge from the pre-training datasets. It is commonly believed that the higher similarity between the pre-training and downstream datasets results in better performance of the downstream task. In addition to the selection of an effective pre-trained model, the customization of the transfer learning model is critically important to the downstream task. This customization is often based on two strategies.

- Fixed feature extractor: Remove the last one or several layers from the pre-trained model, and treat the rest of the pre-trained model as a fixed feature extractor for the downstream dataset. Then, apply a simple classification model over the features from the fixed feature extractor. The training process will only modify the weights of the classification model. The fixed feature extractor strategy can avoid the overfitting problem when the downstream dataset is small.
- Fine-tuning: Replace the last one or several layers of the pre-trained model with customized layers for the downstream task. In the training process, the weights of the pre-trained model are fine-tuned by continuing the back-propagation. In this strategy, the pre-trained model produces generic features, and the fine-tuning process modifies the model to be more specific to the details of the downstream task. The fine-tuning strategy often requires the downstream dataset to be sufficiently large to avoid the overfitting problem.

We explored transfer learning with a fine-tuning strategy for the following reasons: i) the fine-tuning strategy relies more on the data and less on the customization of the network architecture. Specifically, for each pre-trained model, we adopted the same modification strategy, i.e., replacing the last layer with a standard fully-connected (FC) layer and fine-tuning the weights of all layers with the training dataset of the downstream task. ii) We envisioned the downstream dataset is a special task, which requires a different knowledge set from the tasks corresponding to the pre-training dataset. The fine-tuning strategy enables the training using a downstream dataset to customize the model using back-propagation, which puts more emphasis on the newly acquired knowledge. iii) The fixed feature extractor strategies have been explored in literature (Koo et al., 2020; Pompili et al., 2020; Balagopalan et al., 2020).

Koo et al. (2020) and Pompili et al. (2020) employed transfer learning techniques to extract both acoustic and linguistic features from pre-trained models, combined these features with handcrafted features, and customized a convolutional recurrent neural network to perform the downstream tasks. Their customized network architectures though different in detail, produced similar results and conclusions. In comparison, we did not use pre-trained models as a fixed feature extractor, but followed the fine-tuning strategy to train an end-to-end network model. Balagopalan et al. (2020) compared handcrafted features including lexico-syntactic features, acoustic features, and semantic features, with pre-trained automatic features using BERT (Devlin et al., 2018), and concluded that automatic features (83.3% accuracy) outperform the handcrafted features (75.0% accuracy). Edwards et al. (2020) explored multi-scale (word and phoneme level) audio models and their models achieved 79.2% accuracy at best, which is higher than the models using text features (i.e., Word2Vec) and multi-modal fusion. Rohanian et al. (2020) proposed a multi-modal gating mechanism to fusion audio and text features in a Long Short-Term Memory (LSTM) model and achieved a better accuracy of 79.2% compared to the LSTM model with either audio or text features (highest accuracy 73.0%). Yuan et al. (2020) explored disfluencies and fine-tuning pre-trained language models, aligned audio and text using forced alignment, and re-created the punctuation marks in the text using manually-defined thresholds to identify pauses. It achieved an accuracy of 85.4% using BERT and 89.6% using ERNIE (Sun et al., 2020). We consider the thresholds used to identify pauses (Yuan et al., 2020) is still a handcrafted feature. In comparison with the above works, we avoid the complex design and evaluation of handcrafted features and the heavy network architecture. We built an end-to-end network model using the pre-trained networks and a fine-tuning strategy. In addition, Pappagari et al. (2020) employed speaker recognition and natural language processing methods. Specifically, it explored the x-vector (Snyder et al., 2018) and BERT for extracting acoustic and linguistic features, fusioned them with Gradient Boosting Regressor, and achieved 75.0% accuracy using the ADReSS training/test dataset. We considered that our selected pre-training tasks are more representative and similar to the AD classification task, compared to the speaker recognition task (Pappagari et al., 2020; Snyder et al., 2018).

In this paper, we explored a variety of transfer learning techniques and compared several transfer learning models. Note that our training and testing processes strictly followed the ADReSS challenge, i.e., we only used the ADReSS training dataset for training and reported the classification/regression results over the ADReSS testing dataset. Specifically, we investigated the following:

- **Evaluation of transfer learning**: We studied four types of pre-trained models, and customized and fine-tuned our transfer learning models based on the downstream tasks and datasets. We evaluated the impact of the similarity between the pre-training datasets and the downstream datasets on the performance.
- **Multi-modal transfer learning**: We applied a multi-modal transfer learning to incorporate inputs of both audio and text. We investigated whether the audio and text data share complementary information to further improve the performance of the downstream tasks.
- **Multi-task transfer learning**: We applied a multi-task transfer learning to output both the AD/non-AD labels and the Mini-Mental State Examination (MMSE) scores (a test assessing global cognitive functioning). We investigated whether two downstream tasks are highly correlated and whether integrated training can reinforce the performance of the two tasks.

## 2 SPEECH DATASET FOR DEMENTIA RESEARCH

In the ADReSS challenge (Luz et al., 2020), a pre-processed CTP dataset from the Pitt Corpus (Becker et al., 1994) is created with the balanced groups of participants in age, gender, and cognitive status. The ADReSS training dataset includes speech data from 24 male participants with AD, 30 female with AD, 24 male non-AD participants, and 30 female non-AD participants. The ADReSS testing dataset includes speech data from 11 male participants with AD, 13 female with AD, 11 male non-AD participants, and 13 female non-AD participants. The complete dataset information can be found in (Luz et al., 2020). In this paper, we studied the ADReSS dataset, i.e., we trained our models with the ADReSS training dataset and reported the performance of classification and regression tasks over the ADReSS testing dataset.

## 3 PRE-TRAINING DATASETS

In this section, we describe datasets in four domains, i.e., image, audio, speech, and text. These datasets have been successfully explored in their domains for enhanced performance of transfer learning models.

### 3.1 Image dataset

The most commonly used large-scale image classification dataset for pre-training is ImageNet (Deng et al., 2009). ImageNet (http://image-net.org/) is an image dataset organized according to the WordNet hierarchy. Each meaningful concept in WordNet, possibly described by multiple words or word phrases, is called a “synset.” There are more than 100,000 synsets in WordNet, the majority of which are nouns (80,000+). ImageNet provides, on average, 1000 images to illustrate each synset. Images of each concept are quality-controlled and human-annotated. ImageNet pre-training has been widely used in various computer vision tasks, such as fine-grained image classification (Cui et al., 2018; Fu et al., 2017; Russakovsky et al., 2015), object detection (Redmon et al., 2016; He et al., 2017), and sense text detection (Zhou et al., 2017; Wang et al., 2019b).

### 3.2 Audio dataset

AudioSet (https://research.google.com/audioset/) (Gemmeke et al., 2017) is extracted from YouTube videos. It consists of 10-second segments, and each segment is labeled by human effort. All segments are organized in 632 classes, organized in a hierarchical structure with a max depth of 6 levels. AudioSet is considered as a general audio dataset, e.g., the top-level classes include “Human sound,” “Animal sounds,” “Natural sounds,” “Music,” “Sounds of things,” “Source-ambiguous sounds” and “Channel, environment and background.” The dataset contains 1,789,621 segments (4,971 hours) in total. AudioSet is commonly used for the pre-training of acoustic event detection (Arora and Haeb-Umbach, 2017) and sound event tagging (Diment and Virtanen, 2017).

### 3.3 Speech dataset

LibriSpeech (http://www.openslr.org/12/) (Panayotov et al., 2015) is a corpus of approximately 1000 hours of 16kHz read English speech, prepared by Vassil Panayotov with the assistance of Daniel Povey. The data is derived from reading audiobooks from the LibriVox project and has been carefully segmented and aligned. The typical usage of this dataset is for ASR (Zhang et al., 2020; Huang et al., 2020). It could also be used for self-supervised training (Liu et al., 2020; Chi et al., 2020), and transfer to the downstream task like phoneme classification, speaker recognition, and sentiment classification.

### 3.4 Text dataset

BERT (https://github.com/google-research/bert) dominates Natural Language Processing (NLP) research by learning powerful and universal representation and utilizing self-supervised learning at the pre-training stage to encode the contextual information. The representation is beneficial to performance, especially when the data of the downstream task is limited. The pre-training datasets for BERT include the BooksCorpus (Zhu et al., 2015) (800M words) derived from textbooks and Wikipedia (2,500M words) derived from Wikipedia websites. BERT (Devlin et al., 2018) and its variants (Beltagy et al., 2020; Lan et al., 2019; Liu et al., 2019) have been developed using self-supervised training for downstream tasks, e.g., text classification and question answering. Longformer (Beltagy et al., 2020) is a variant of BERT to allow the model to learn long dependencies in pre-training, and its pre-training databases additionally include one-third of a subset of the Realnews dataset (Zellers et al., 2019) with documents longer than 1,200 tokens as well as one-third of the StoryCorpus (Trinh and Le, 2018).

## 4 DEEP TRANSFER LEARNING MODEL

Our transfer learning models were built within three steps, 1) **pre-training**, 2) **fine-tuning**, and 3) **testing**. In the pre-training step, a model was trained with a large-sized dataset. In the fine-tuning step, we tuned the model with the ADReSS training dataset. In the testing step, we evaluated the model using the ADReSS testing dataset. In the following, we introduce the transfer learning models based on two pre-training approaches: a *supervised classification* approach and a *self-supervised learning* approach.

### 4.1 Supervised classification approach - MobileNet and YAMNet

For this approach, we explored the audio part of the ADReSS datasets. We observed the ADReSS organizers segmented the audio data into small pieces by setting the log energy threshold parameter to 65dB with a maximum duration of 10 seconds from (Haider et al., 2019; Luz et al., 2020). However, there was a concern that the segmentation may cause critical time-series information loss. Any smaller speech segments hardly represent the overall speech sample. In addition, the speech continuity is removed by segmentation, making the model inaccurately capture the time-series characteristics. Thus, our approaches aimed to accommodate an entire speech sample of each participant as input and preserve the time-series characteristics of the speech, similar to works (Hershey et al., 2017; Zhang et al., 2018).

**Figure 1.**
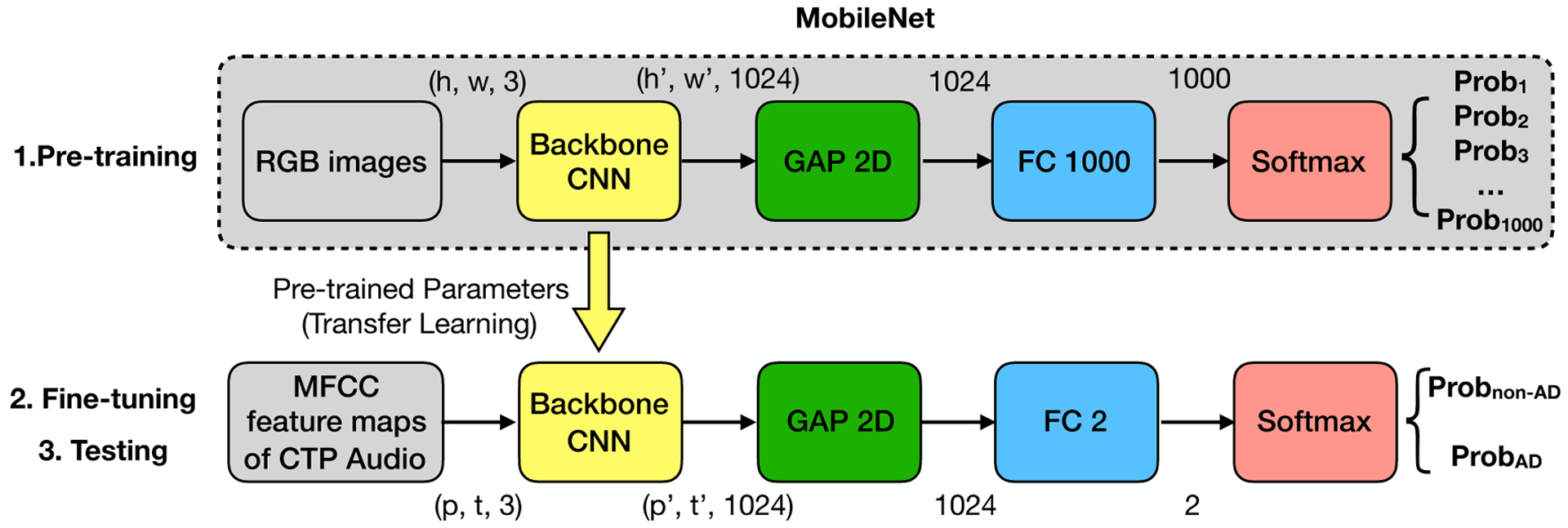
Supervised Classification Approach

*MobileNet* is a lightweight network architecture that significantly reduces the computational overhead as well as parameter size by replacing the standard convolution filters with the depth-wise convolutional filters and the point-wise convolutional filters, proposed by (Howard et al., 2017). The total parameters of the MobileNet backbone are of a size 17.2 MB, significantly less than other convolutional neural networks. Considering the limited size of the speech dataset, we considered a smaller model with less complexity, such as MobileNet, which may worth being tested. MobileNet is pre-trained with the ImageNet dataset for an image classification task. The MobileNet architecture is shown at the above layer of the Figure 1. With an RGB image as input, the output is the probability that the image belongs to each of the 1000 classes.

*MobileNet architecture.* The core of MobileNet architecture is a backbone Convolutional Neural Network (CNN), which consists of a set of convolution, pooling, and activation operations. The detailed architecture can be found in the paper (Howard et al., 2017). We used the full width (1.0) MobileNet backbone pre-trained on a resolution of 128*128 images. The backbone takes an image as an input, which is 3-dimensional (*h, w,* 3)-matrix where *h* is height, *w* is width, and 3 represents the RGB channel. The backbone converts an input of (*h, w,* 3)-matrix to an output of (*h′, w′,* 1024)-matrix where (*h′, w′*) are functionally related to (*h, w*), and 1024 represents the feature channel number, i.e., the depth of the backbone CNN. The output (*h′, w′,* 1024)-matrix is then fed to a Global Average Pooling (GAP) layer for reducing the dimensions of *h′* and *w′* and obtaining a 1024-dimension feature. A Fully Connected (FC) layer with 1000 neurons produces the output according to the wanted 1000 classes. Lastly, a softmax activation layer is added to produce the classification results as the probabilities for 1000 classes that add up to 1.

#### Transfer learning via MobileNet.

MobileNet is pre-trained for an image classification task where its input is an image, and its output is probabilities of the classes. To apply transfer learning of MobileNet to our AD classification task, in the fine-tuning and testing steps, we need to convert an audio sample to an image sample and customize the model for the AD/non-AD outputs.

##### 1. Extracting Mel Frequency Cepstral Coefficient (MFCC) feature maps from audio samples.

Mel-frequency cepstral coefficients have been widely used in speech recognition research (Muda et al., 2010). Yancheva and Rudzicz (2016); Fraser et al. (2016) carried out an acoustic-prosodic analysis on the Pitt Corpus using 42 MFCC features. We extracted an MFCC feature map for each participant’s entire speech sample. The MFCC feature map is denoted as a (*p, t*)-matrix where the hyper-parameter *p* (64) is the MFCC order, and *t* is related to the duration of the speech sample. We used the librosa function with a sampling rate of 22050, a window size of 2048, and a step size of 512. By extracting the MFCC feature maps, we converted the speech dataset to an image dataset. The advantages of MFCC feature maps include conversion from speech to MFCC feature maps can be done automatically; the silent pauses in the audio data were preserved as a distinctive feature in MFCC feature maps; and speech from the investigator and filled pauses from the participant were preserved in MFCC feature maps and shown to be important (Tóth et al., 2018). While identifying these audio segments requires expensive human efforts or customized ASR, we envision the classification model with the input of the MFCC feature maps may learn and understand the patterns of the information.

##### 2. Customizing model for the downstream task.

Our proposed model is shown at the bottom layer of the Figure 1. Our architecture employs the pre-trained backbone CNN module from the MobileNet. Denote the MFCC feature map of the audio sample as a (*p, t,* 1)-matrix. To match with the module input, i.e., an RGB image, we duplicated the MFCC feature map twice and made the MFCC feature map as a (*p, t,* 3)-matrix. In this way, we can feed the MFCC feature map into the backbone CNN module of the MobileNet in the same way as an RGB image. The output of the backbone CNN is denoted as a (*p′, t′,* 1024)-matrix where (*p′, t′*) are functionally related to (*p, t*). We employed a GAP-2D (two-dimensional) to reduce *p′* dimension and *t′* dimension of the matrix. We then employed a fully-connected layer and a softmax activation layer to produce the classification results as two probabilities for the two classes AD/non-AD that add up to 1.

#### Transfer learning via YAMNet.

While the MobileNet architecture is pre-trained with the ImageNet dataset, Gemmeke et al. (2017) pre-trained a similar architecture using the AudioSet dataset, called YAMNet. The input of YAMNet is the Mel spectrogram from audio data with dimensions of (*p, t,* 1). Compared to MobileNet, YAMNet might better apply to our downstream task because the pre-training dataset and the downstream dataset are both audio datasets; and the input formats to the Backbone CNN in the pre-training/fine-tuning/testing phase are kept the same, i.e., a feature vector of (*p, t,* 1).

### 4.2 Self-supervised learning approach - BERT

**Figure 2.**
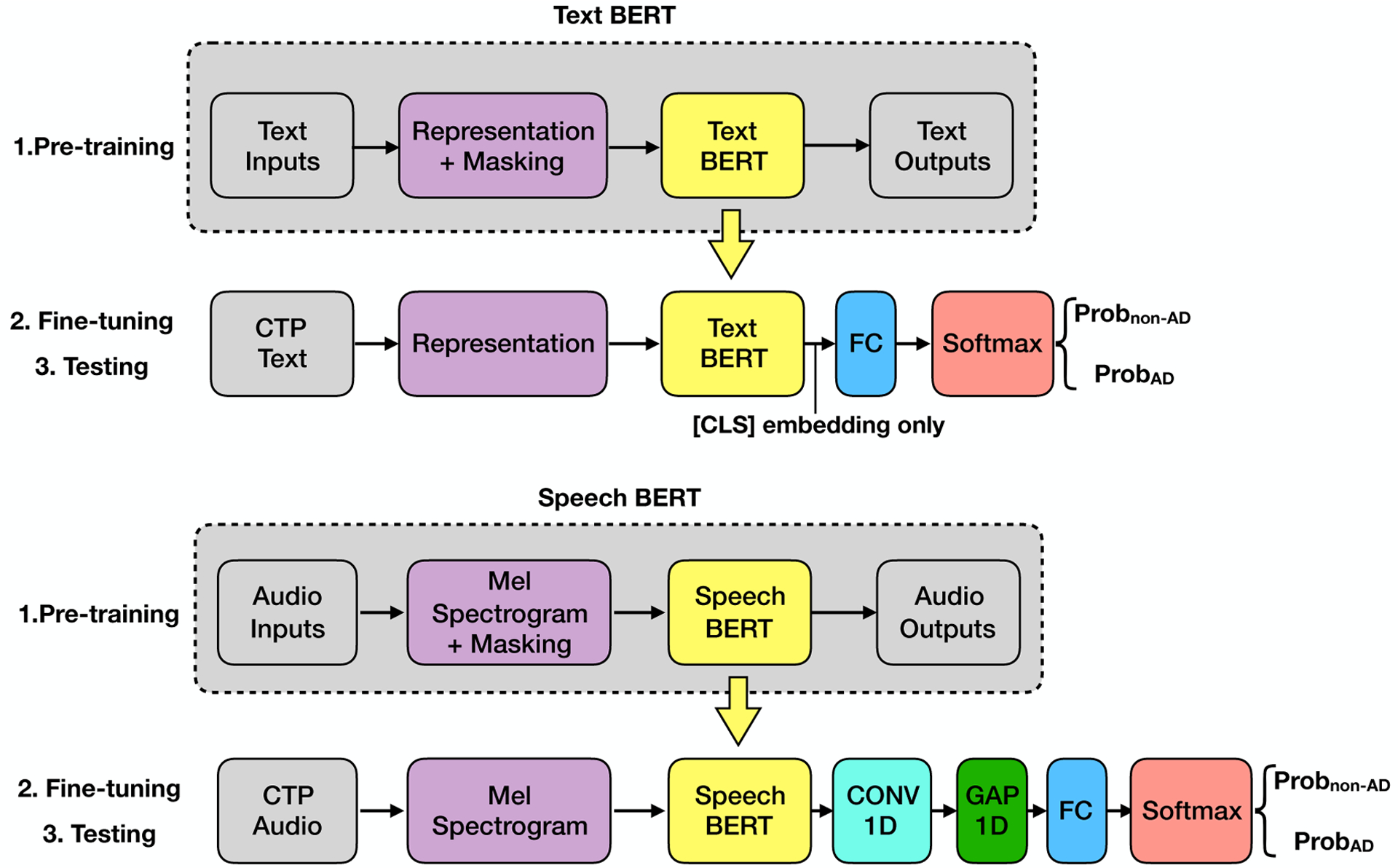
Text BERT and Speech BERT

While the supervised classification approach utilizes labeled datasets, self-supervised learning approaches take advantage of unlabeled datasets for pre-training. The removal of the labeling requirement enables the model to extract knowledge from an extended range of data sources, e.g., digital books, and Wikipedia, and online news. We propose a Text BERT model and a Speech BERT model for AD classification, as shown in Figure 2.

#### Transfer learning via Text BERT.

BERT (Devlin et al., 2018) is a milestone in the natural language processing domain. BERT is pre-trained with BooksCorpus (Zhu et al., 2015) (800M words) and Wikipedia (2,500M words). It adopts two self-supervised tasks in the **pre-training** step: Masked Language Model (MLM) and Next Sentence Prediction (NSP). Specifically, given a pair of sentences, we first put a special [CLS] token at the beginning of the first sentence and a special [SEP] token between two sentences. Secondly, random masking is applied to mask a set of words with a special [MASK] token. Then the pre-processed input is fed into the BERT model, which then outputs an embedding corresponding to each input token. The pre-training is performed via the two self-supervised tasks: the MLM task aims to predict the masked words with the context; the NSP task aims to predict whether the second sentence is followed by the first sentence in the original dataset. In the **fine-tuning** and **testing** steps, the output embedding of the [CLS] token is used. To apply BERT to our AD classification task, we added a fully connected (FC) layer and a softmax activation layer to the output of the BERT model. The FC layer has two neurons, which stands for the AD and no-AD classes, respectively.

#### Transfer learning via Speech BERT.

The Speech BERT, named Mockingjay (Liu et al., 2020), is similar to the Text BERT except for some differences: The input is the Mel spectrogram of speech data instead of the word embeddings. The pre-training task contains only the Masked Acoustic Model (MAM) task. The input does not have the [CLS] and other special tokens. Thus, instead of using output embedding of the [CLS] token for classification, we used output embeddings of all the tokens. To apply Speech BERT to our AD classification task, the output of the Speech BERT is fed into a 1D convolutional layer that convolutes through time dimension, then fed into a global average pooling layer to obtain the average through time dimension, and finally fed into an FC layer and a softmax activation layer.

## 5 MULTI-MODAL TRANSFER LEARNING

**Figure 3.**
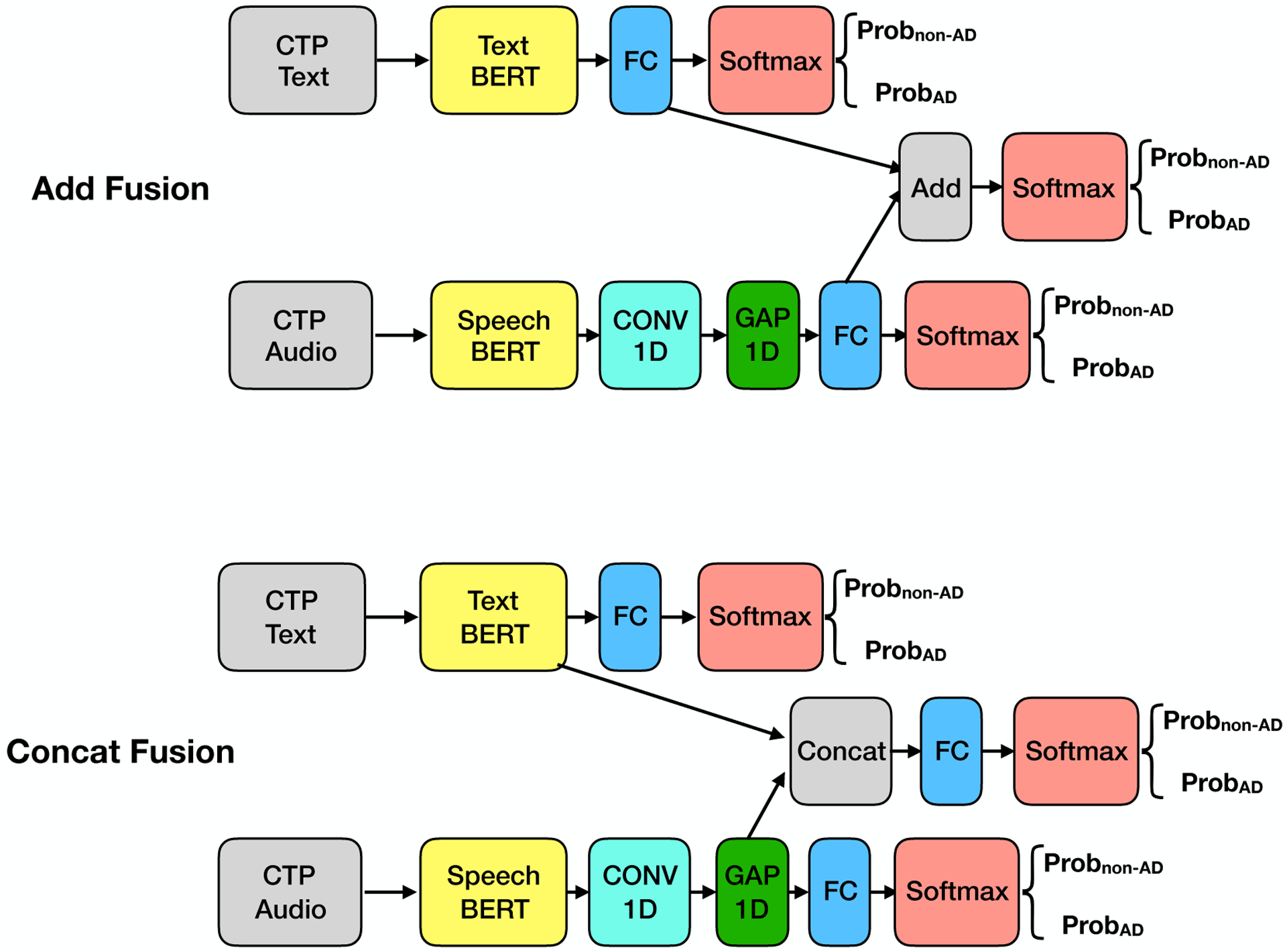
Multi-modal transfer learning using Text/Speech BERT (Dual BERT)

While Text BERT and Speech BERT models analyze text and audio datasets separately, we explored a multi-modal transfer learning via a Dual-BERT model, using both text and audio as inputs. We envision that the text and audio data of a given patient are highly related, and the outputs could reinforce each other during the training process. Dual-BERT incorporates two pre-trained BERT models, one is Text BERT, and the other is the Speech BERT. As shown in Figure 3, the architectures of the Speech BERT and the Text BERT models remain the same as in the previous section. We further designed two types of fusion methods, Add fusion and Concat fusion. We used term “training” instead of “fine-tuning” in the following, as we mainly considered the new multi-modal transfer learning. For each fusion method, we also considered two types of training strategies, separate training and joint training.

### Add fusion model.

The outputs of our previous models are probabilities from the last softmax activation layer. Thus, we considered an Add fusion that adds up the outputs of the FC layers of two models, as shown in the upper part of Figure 3. If the Text BERT and Speech BERT models have consistent classification results, the Add fusion model outputs the result with more confidence compared to any of the two single models. On the other hand, if the two models have inconsistent classification results, the Add fusion model outputs the result that receives higher confidence from any of the two models. We considered two training strategies. 1) (Separate) We train the Text BERT and Speech BERT with text and audio, respectively. Then, the Add fusion layer will only be considered during the testing process. 2) (Joint) We train the Text BERT and Speech BERT jointly using the joint output from the Add fusion layer. The difference between these two training strategies is that the first strategy considers the confidence of the models, while the second one further considers the complementary information between text and audio data. The Add fusion part has no trainable parameters. In the separate training strategy, the training does not apply to the Add fusion part; in the joint training strategy, the Add fusion part is involved in the training process but has no parameters to be learned.

### Concat fusion model.

Another way to explore the multi-modal transfer learning is to concatenate the tensors of the Text BERT and Speech BERT models before the FC layer. As shown in the bottom part of Figure 3, after the concatenation, the Concat fusion model has an FC layer with two neurons for classification of AD/non-AD. In this model, features from text and audio are better integrated for the classification task. The Concat fusion model always requires joint training for the additional FC layer. We have two training strategies. 1) (Separate) We train the Concat fusion model using three outputs separately. 2) (Joint) We train the Concat fusion model using the joint output only.

## 6 MULTI-TASK TRANSFER LEARNING

Multi-task transfer learning aims to solve multiple learning tasks at the same time while exploiting commonalities and differences across tasks. This can result in improved learning efficiency and enhanced performance for the task-specific models when compared to training the models separately.

**Figure 4.**
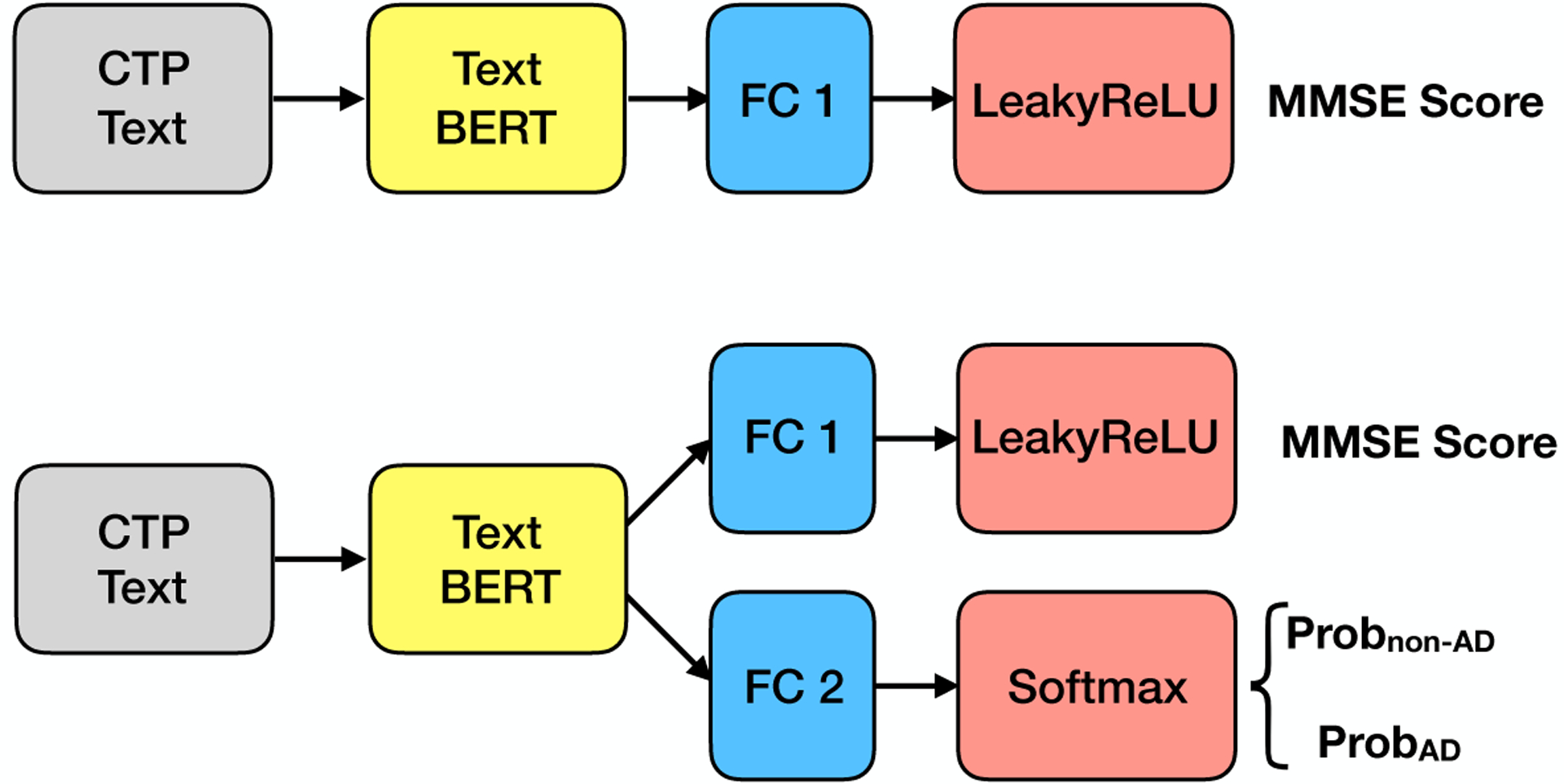
Multi-task learning using Text BERT

The ADReSS challenge provides both AD/non-AD labels and MMSE scores for each data sample. In this section, we focused on the Text BERT as it produces significantly better results than the Speech BERT. As shown in the upper part of Figure 4, we first applied transfer learning from the Text BERT to an MMSE regression task; we placed an FC layer with a single neuron to the output of the Text BERT, and then added a Leaky ReLU layer to output the MMSE score. Since the MMSE scores are non-negative values, we adopted the Leaky Rectified Linear Unit (ReLU) activation and the mean squared error loss. The bottom figure in Figure 4 shows a multi-task transfer learning where we put an FC layer with a single neuron for the regression task and an FC layer with two neurons for the classification task. The classification task employs the softmax activation layer, and the regression task employs the Leaky ReLU activation layer. For loss functions, the classification task uses the cross-entropy loss, and the regression task uses the mean squared error loss. For training, we jointly optimized the cross-entropy loss and the mean squared error loss with the corresponding labels.

## 7 PERFORMANCE EVALUATION

In this section, we provide a comprehensive evaluation of the proposed deep transfer learning models. We strictly followed the ADReSS challenge (Luz et al., 2020), using the ADReSS training and testing datasets.

### 7.1 Implementation details

We followed the original implementation of the pre-trained models. Specifically, the speech BERT and text BERT were implemented with PyTorch. The MobileNet and YAMNet were implemented with Tensorflow. We downloaded the pre-trained parameters of these models from online sources. For the classification task (AD/non-AD), we used the cross-entropy loss, and for the regression task (MMSE), we used the mean squared error loss. We trained our models using the Adam algorithm as optimizer (Kingma and Ba, 2014) with batch size 8 and a small learning rate of 1e-6 for models that do not use Speech BERT. For models that use Speech BERT, as our Graphics Processing Unit (GPU) resource has 32GB memory (NVIDIA TESLA V100), we used batch size 1 to adapt our training process to the limited memory resources. We employed a fine-tuning strategy and trained all layers, including those in the pre-trained models.

### 7.2 Training strategy

Our training strategy for all models had five rounds. In each round, we used the ADReSS training dataset to train a model with a maximum of 2000 epochs. The training stopped before reaching 2000 epochs only if the training loss was less than a pre-defined threshold of 1e-6. After the training, we selected the epoch with the smallest training loss and obtained the performance result over the ADReSS testing dataset using the selected epoch. We repeated the above process for five rounds, obtained five results, and reported their *mean and standard deviation*. We consider that the *mean and standard deviation* represent the effectiveness of the model. We also reported the Best result among all epochs in five rounds to reveal the maximum potential of the models.

### 7.3 Evaluation metrics

For the classification task, we employed evaluation metrics of accuracy$\frac{TN+TP}{N}$, precision$\pi =\frac{TP}{TP+FP}$, recall$\rho \;=\;\frac{TP}{TP\;+\;FN}$, and F1 score$\frac{2\pi \rho }{\pi +\rho }$, where *N* is the number of participants, *TP* , *FP* and *FN* are the numbers of true positives, false positives and false negatives, respectively. For the regression task, we employed Root-Mean-Square Error (RMSE), the same metric used in the baseline paper provided by the ADReSS challenge.

### 7.4 Evaluation of deep transfer learning models

In this subsection, we reported the performance results of our transfer learning models with an input of audio data or text data. **MobileNet**, **YAMNet**, and **Speech BERT** were pre-trained with ImageNet, AudioSet, and LibriSpeech datasets, respectively, and were used to analyze CTP audio data. **BERT base** and **BERT large** were pre-trained with BooksCorpus, Wikipedia, and **Longformer** were pre-trained with additional Realnews and StoryCorpus. They were used to analyze CTP text data. To show the advantage of transfer learning, we also reported the performance results of the models without pre-training. The performance results are shown in Table 2.

#### MobileNet.

The classification accuracy of MobileNet is 59.00 ± 5.66% without pre-training or 58.8 ± 3.49% with pre-training. Both MobileNet models achieved low accuracy, and the pre-training process surprisingly lowered the performance. We concluded the main reason is the knowledge difference between the pre-training image dataset and the CTP audio dataset. However, we found that the pre-training helped produce stable results with a lower standard deviation (from 5.66% to 3.49%). In addition, we found that Best accuracy reaches 77.08% with pre-training, much higher than 72.91% without pre-training. In other words, the model with pre-training has the potential to achieve higher accuracy, but the model cannot be fine-tuned to the optimal status due to the limited downstream dataset.

#### YAMNet.

In general, YAMNet would be more effective than the MobileNet for our downstream task because the pre-training dataset in YAMNet is AudioSet, which is more similar to the CTP audio dataset. We confirmed this conjecture with our evaluation results of YAMNet. The classification accuracy of YAMNet without pre-training is 53.8 ± 6.88%, and the accuracy of YAMNet with pre-training is increased to 66.2 ± 4.79%. The YAMNet with pre-training resulted in a significant improvement of 12.4% compared to the same model without pre-training, which demonstrates the similarity between the AudioSet and the CTP audio dataset. In addition, the pre-training enabled the YAMNet to produce more stable outputs (from 6.88% to 4.79%) and higher Best accuracy (from 79.17% to 83.33%).

#### Speech BERT.

Speech BERT, similar to Text BERT, employs a self-supervised learning approach. The pre-training process employs the Masked Acoustic Model (MAM) task. Speech BERT has a length restriction problem of max positional encoding in pre-training of 5000 tokens (about 1 minute). To solve this problem, in training, if the audio sample produces more than 5000 tokens, we randomly choose a window to sample the audio for 5000 tokens. And in the testing, we used a non-overlapped sliding window technique to sample the whole audio and averages the classification probabilities corresponding to all windows. We further filtered the audio data of the investigator to reduce the audio length, while for MobileNet/YAMNet, both audio data of the investigator and participant were kept as input.

We observed that the Speech BERT model with pre-training resulted in less accuracy 63.33%, compared to 66.67% from the model without pre-training. This finding may have been due to the Speech BERT models employing a self-supervised MAM task, which is significantly different from our downstream task (i.e., classification). Alternatively, the self-supervised MAM task aims to explore the strong correlation between the audio segments. While such a correlation in the transcript is explicit due to the language model, the correlation among audio segments might be more complicated and more challenging to be learned. In addition, the pre-training process helps to increase the potential of the model by providing a higher Best accuracy of 79.17% (> 77.08% without pre-training).

#### Text BERT.

We considered three Text BERT models, i.e., BERT base and BERT large (Devlin et al., 2018), and Longformer (Beltagy et al., 2020). The BERT base model has 12 Transformer encoders, and the BERT large model has 24 Transformer encoders. While the BERT base and BERT large were pre-trained with a max length of 512 tokens, the Longformer were pre-trained with a max length of 4096 tokens. Therefore, when our text sample from ADReSS datasets is converted to be larger than 512 tokens, truncation is required in the BERT base and large models. In the Longformer model, all text samples from ADReSS datasets can be encoded within 4096 tokens, and thus truncation is not needed. In addition, the pre-training databases of Longformer additionally include longer text samples from Realnews and StoryCorpus. To adapt the ADReSS text dataset to the Text BERT models, we removed the symbols that do not appear in the pre-training dataset but appear in the ADReSS text dataset.

We found the performance results of all Text BERT models are better than the previous models on audio data. Without pre-training, BERT base achieved 76.67%. With pre-training, BERT base achieved 80.83%, BERT large achieves 81.67%, and Longformer achieves 82.08%. The corresponding Best accuracy increased from 81.25% (BERT base without pre-training) to 85.42% (BERT base), 87.50% (BERT large), 89.58% (Longformer). These findings suggest that the Text BERT models show significantly better performance because of the similarity of the pre-training text dataset and the CTP text dataset. In addition, the Longformer resulted in improved performance because it supports the input of longer text samples without truncation and has been pre-trained with additional similar datasets.

### 7.5 Evaluation of multi-modal transfer learning

Focusing on evaluating multi-modal transfer learning, we expected the joint training using both audio data and text data to improve the performance results of previous models. In Table 3, we list the performance results of ten models. The first one is BERT base, and the second one is Speech BERT, which was evaluated in the previous section. Their performance results will serve as a baseline. The next seven models are variants of the Dual BERT models. Their architectures are a combination of a Speech BERT model and a Text BERT model. As discussed in Section 5, Dual BERT can employ the Add fusion or the Concat fusion to combine the Speech BERT and the Text BERT, and can be trained with a separate training strategy or a joint training strategy. The last multi-modal transfer learning replaced Speech BERT with YAMNet as YAMNet achieves an accuracy (66.2%) higher than Speech BERT (63.33%).

The following observations were made:

- All seven Dual BERT models achieved higher classification accuracy than the two baseline models, confirming that the text data and audio data have complementary information that can be jointly learned by the model for improved performance.
- Concat fusion achieved higher classification accuracy than Add fusion. While the Add fusion picks one model with higher confidence in the classification results, the Concat fusion aims to merge the features of both text data and audio data for a hybrid representation. The performance gain of the Concat fusion further confirms the complementary information between the text data and audio data.
- From the previous analysis, we found the Speech BERT without pre-training achieved a higher accuracy (66.67%) than the Speech BERT with pre-training (63.33%). Thus, we evaluate a multi-modal transfer learning model using the Speech BERT without pre-training and BERT base with pre-training. As shown in Table 3, we confirm that the pre-training of Speech BERT helps the multi-modal transfer learning to achieve a higher accuracy (82.50%), compared to the Dual BERT without pre-training on speech model (82.08%).
- From the previous analysis, we found BERT large (81.67%) and Longformer (82.08%) outperformed BERT base (80.83%). Thus, we replaced BERT base with BERT large and Longformer in the Dual BERT. While the multi-modal transfer learning using BERT large achieved the highest accuracy (82.92%), the multi-modal transfer learning using Longformer achieves the highest Best accuracy (89.58%).
- From the previous analysis, we found the YAMNet yielded the highest accuracy result (66.20%) among all the models using audio data. Thus, we evaluated a multi-modal transfer learning using the YAMNet and BERT base. However, this model did not outperform any of the Dual BERT models.

### 7.6 Evaluation of multi-task transfer learning

#### Relation between MMSE regression and AD classification.

**Figure 5.**
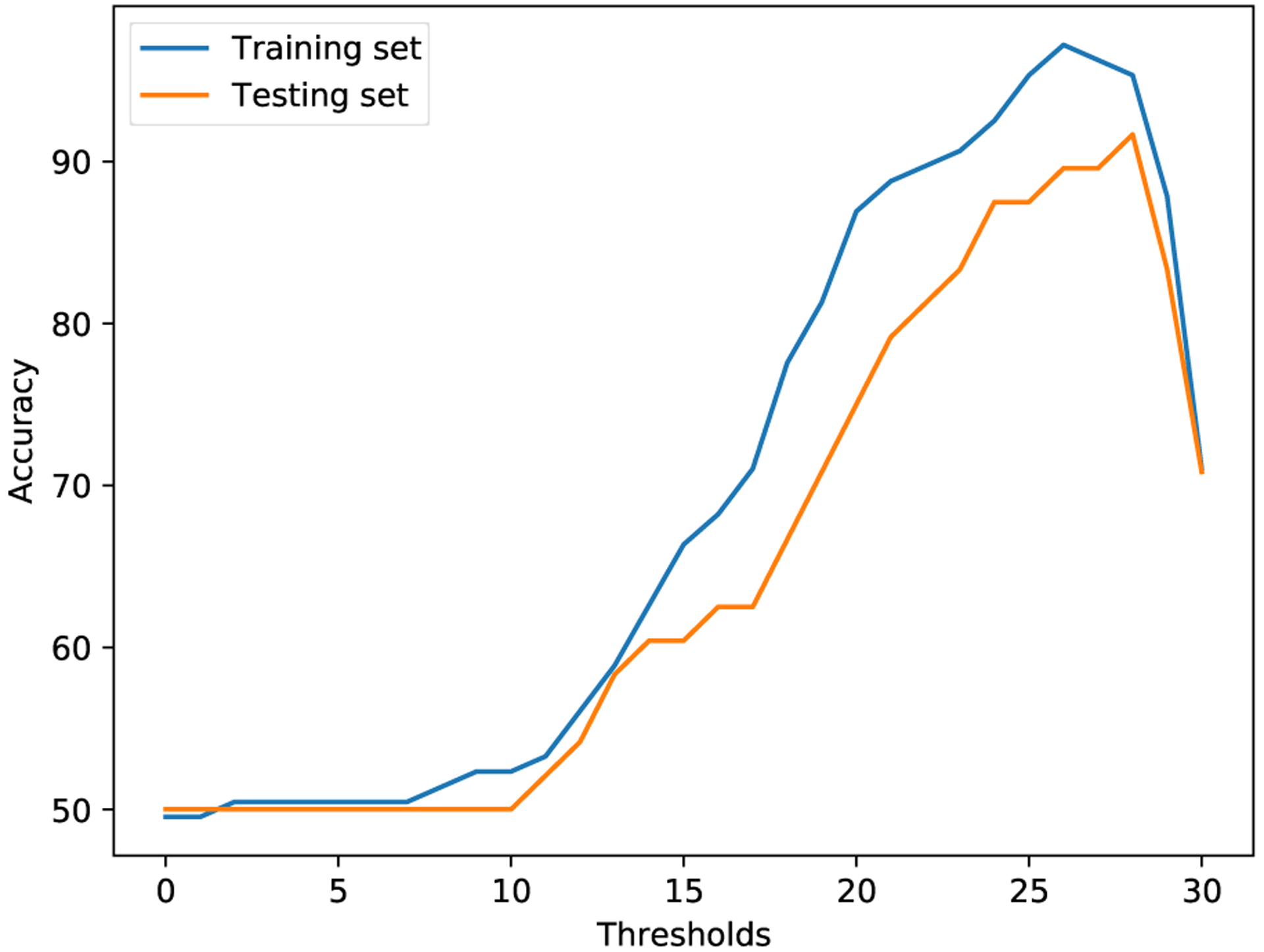
Threshold-based strategy (0–30)

Given the ADReSS dataset, we explored a threshold-based strategy to understand the relation between the MMSE scores and AD/non-AD labels. We set a threshold *T* on MMSE scores to infer AD/non-AD status. If a patient’s MMSE score is less than *T*, the patient’s data is labeled with AD; if a patient’s MMSE score is larger or equal to *T*, the patient’s data is labeled with non-AD. We reported the performance result of the threshold-based strategy over the ADReSS training/testing dataset separately in Figure 5 and Table 4. We found that for the ADReSS training dataset, the highest accuracy is 97.2% at a threshold of 26, and for the ADReSS testing dataset, the highest accuracy is 91.67% at a threshold of 28. If we adopt the threshold of 26 from the training dataset and apply it to the testing dataset, the threshold-based strategy results in an accuracy of 89.58%, which is the upper bound that multi-task transfer learning theoretically can achieve. According to the CTP dataset description (Becker et al., 1994), the patients with AD have an MMSE score range 8–30, while the patients with non-AD have a range 26–30. The AD labels are determined from seven cognitive domains, including memory, construction, perception, attention, language, orientation, and executive functions. In comparison, the MMSE is a 30-point widely used cognitive screening measure, taking about 10 minutes to administer. In our evaluation, given the limited number of data samples, a small number of inconsistent cases might produce a negative impact on the joint training process when the outputs of single-task models are highly consistent with the corresponding labels/scores.

We focused on evaluating the proposed multi-task transfer learning, which is built on the BERT base model with an input of the CTP text data. One challenge of the multi-task transfer learning model is the imbalanced loss from the AD classification task and the MMSE regression task. Denote the regression loss (mean squared error) as *l*_*mse*_ and the classification loss (cross-entropy) as *l*_*ce*_. We define the total loss of the multi-task transfer learning model as *l* = *λl*_*mse*_ + *l*_*ce*_, where *λ* is a balance factor to avoid the unbalanced impact between the classification loss and regression loss. In our experiment, we set *λ* = 0.01.

We evaluated a regression model and a multi-task transfer learning model using BERT base. As shown in Table 5, when using BERT base without pre-training, the multi-task transfer learning model outperformed the single-task models, i.e., the classification accuracy is increased from 76.67% to 78.75%, and the RMSE decreased from 5.18 to 4.70. The evaluation results confirmed that the two tasks help each other to achieve a better performance, especially when both single-task models have room to be improved. In comparison, when using BERT base with pre-training, the multi-task transfer learning model introduced limited performance gain in classification and introduced a negative impact in the regression model. Specifically, the average classification accuracy remained the same at 80.83%, the standard deviation decreased from 2.04% to 1.56%, and Best accuracy is increased from 85.42% to 87.50%, close to the accuracy of 89.58% from the threshold-based strategy. For classification, multi-task learning kept the training more stable and increased the maximal potential of the model, and the MMSE scores provide a limited positive impact on the AD classification task. For regression, RMSE increased from 4.15 to 4.96, which reveals a negative impact of the joint training. This may have been due to the inconsistent cases of MMSE scores and AD/non-AD labels, and the MMSE regression task is more fined-grained and thus received a stronger impact from the inconsistent cases.

### 7.7 Summary of best cases using transfer learning

Table 6 shows the best cases of our experiments of text-based, audio-based, and multi-modal transfer learning models. The best case of the audio model achieved 66.20%, while the best case of the text model achieved 82.08%. We consider the performance gain of the text model may be due to the high similarity between the pre-training text dataset and the CTP text dataset. In addition, the multi-modal model using both audio and text achieved the highest accuracy of 82.92% in its best case, demonstrating that audio and text data provided complementary information. Our multi-task model achieved an accuracy of 80.83%, lower than the accuracy of the text-based model and the multi-modal model. We consider the performance degradation of the multi-task model may be due to the inconsistency between labels and scores that were used in multiple tasks.

## 8 CONCLUSIONS

We explored transfer learning techniques for an AD classification task and an MMSE regression task. The transfer learning models were pre-trained with general large-sized datasets, and fine-tuned and tested using the ADReSS datasets. Our models had minimal customization and mostly relied on the training data and fine-tuning process to incorporate the knowledge of the downstream task into the pre-trained model. From our comprehensive evaluation, we drew the following three conclusions.

### Transfer learning on text data results in high accuracy, but transfer learning on audio data might have more potential.

Our findings showed that the transfer learning on text data achieved high accuracy in the downstream tasks and always outperformed the transfer learning on audio data. This suggests that the transfer learning model understands the text better than the audio. We considered the text data is generated from the audio data through human transcribing effort. Thus, the additional information that the text data contains, but not the audio data contains, might be the transcriber’s knowledge in the transcribing process. The transcriber extracts task-specific information, such as the CTP and information units in the photo. However, while the text data implicitly contains the transcriber’s knowledge, the audio data does not have. And our training process of the transfer learning models on audio data does not take advantage of the transcriber’s knowledge. We expect that the task-specific information is highly useful, and our transfer learning models on audio data can be further improved by integrating such information. In addition, different parts of the text might be highly relevant, but the relevance of different audio segments might be unclear and difficult to be learned by the proposed models. Thus, we concluded that the low accuracy of the transfer learning on audio data was likely observed because the introduced pre-trained models did not extract good representation from the audio data from the downstream perspective. However, we envision that the future large-sized speech datasets might contain audio data and auto-translated text data via ASR. For example, the larger CTP dataset WLS (Herd et al., 2014) contains text data from Kaldi ASR. Thus, our future work on transfer learning aims to explore a better pre-trained model, including supervised ASR models and self-supervised audio models.

### Multi-modal transfer learning reveals complementary information of text and audio.

Our multi-modal transfer learning introduced a slight but not significant improvement in terms of accuracy, demonstrating that the audio and text data provide complementary information. Specifically, while the text model alone already achieved high accuracy, adding the analysis of audio data can improve performance results almost in every case. More importantly, if we consider that the text data contains semantic information only, the complementary information that the audio data contains, but not the text data contains, might be the non-semantic information, such as filled pause, silent pause, and other implicit features. The non-semantic information may or may not be used to implement effective classification alone, but they should be useful if they are jointly analyzed with the semantic information. We envision that the model can be improved if it learns the positional information of both semantic and non-semantic features, e.g., the pause information between words or between sentences.

### Multi-task transfer learning reveals positive and negative impacts on AD classification and MMSE regression.

Our multi-task transfer learning of the classification and regression tasks yielded significantly better performance when both single-task models did not perform well. The performance gain is obtained due to the consistency between most MMSE scores and the AD/non-AD labels. However, when the outputs of the single-task models are highly consistent with the corresponding labels/scores, the performance of multi-task learning declined due to a small number of samples with inconsistent scores and labels. This suggests the need to investigate the meaning behind the AD classification task and the MMSE regression task. The AD/non-AD labels seem coarse-grained, but they are generated by evaluating patients on several cognitive domains. The MMSE is less accurate and considered a screening measure of global cognitive functioning. We confirmed that such inconsistency existed by exploring a threshold-based strategy on the ADReSS training and testing datasets. Thus, we considered that multi-task transfer learning produces a limited impact on accuracy improvement due to the inconsistency between labels and scores. In conclusion, we believe that the deep transfer learning techniques need to be simple, comparable, and applicable to newer tasks, larger datasets, and heterogeneous labels to produce a long-lasting impact in dementia research.

## Figures and Tables

**Table 1.** Cookie Theft Picture Datasets

Dataset	Language	Total	HC	MCI	AD
ADReSS (Luz et al., 2020)	English	156	78		78
Pitt Corpus (Becker et al., 1994)	English	312	104		208
WLS (Herd et al., 2014)	English	1366			
IVA (Mirheidari et al., 2019b)	English	33	16		17
Hebrew CTP (Kavé and Dassa, 2018)	Hebrew	70	35		35
MECSD (Wang et al., 2019a)	Mandarin	85	65		20
NTU (Chien et al., 2019)	Mandarin	50	40		10
Swedish CTP (Wallin et al., 2016)	Swedish	67	36	31	
French CTP (Fraser et al., 2019b)	French	58	25		33

**Table 2.** AD Classification results using audio or text and with or without pre-training. AD: Alzheimer’s Disease. Accuracy: mean and standard deviation of results of 5 rounds. Best: highest accuracy of all epochs in 5 rounds.

Model	Pre-training dataset	Classes	Precision %	Recall %	Fl %	Accuracy %	Best %
Audio (Luz et al., 2020)	-	non-AD	67	50	57	62	
		AD	60	75	67		
MobileNet	-	non-AD	60.40 ± 7.86	58.40 ± 22.76	56.20 ± 13.79	59.00 ± 5.66	72.91
		AD	61.40 ± 6.89	59.80 ± 21.07	57.60 ± 10.54		
	ImageNet	non-AD	72.80 ± 6.97	28.00 ± 8.15	40.40 ± 9.85	58.80 ± 3.49	77.08
		AD	55.80 ± 2.48	90.40 ± 1.96	69.00 ± 1.67		
YAMNet	-	non-AD	52.20 ± 11.74	19.80 ± 22.61	24.60 ± 22.81	53.80 ± 6.88	79.17
		AD	53.40 ± 5.95	87.60 ± 9.56	65.80 ± 1.33		
	AudioSet	non-AD	69.60 ± 6.80	59.20 ± 7.73	63.40 ± 5.57	66.20 ± 4.79	83.33
		AD	64.40 ± 3.93	73.40 ± 8.82	68.60 ± 4.84		
Speech BERT	-	non-AD	67.74 ± 3.69	64.17 ± 3.34	65.82 ± 2.68	66.67 ± 2.95	77.08
		AD	65.84 ± 2.43	69.16 ± 5.65	67.39 ± 3.71		
	LibriSpeech	non-AD	66.13 ± 4.12	55.00 ± 4.86	59.94 ± 3.78	63.33 ± 3.12	79.17
		AD	61.48 ± 2.76	71.67 ± 4.86	66.12 ± 3.08		
Text (Luz et al., 2020)	-	non-AD	70	87	78	75	-
		AD	83	62	71		
BERT base	-	non-AD	78.12 ± 1.98	74.17 ± 3.12	76.05 ± 1.82	76.67 ± 1.56	81.25
		AD	75.47 ± 2.08	79.17 ± 2.63	77.23 ± 1.50		
	BooksCorpus/Wiki	non-AD	78.46 ± 1.89	85.00 ± 2.04	81.60 ± 1.96	80.83 ± 2.04	85.42
		AD	83.64 ± 2.22	76.67 ± 2.04	80.00 ± 2.13		
BERT large	BooksCorpus/Wiki	non-AD	83.05 ± 5.12	80.00 ± 3.12	81.40 ± 3.09	81.67 ± 3.34	87.50
		AD	80.65 ± 2.66	83.33 ± 5.89	81.89 ± 3.64		
Longformer	BooksCorpus/Wiki/ Realnews/Stories	non-AD	77.87 ± 3.75	90.00 ± 2.04	83.44 ± 2.33	82.08 ± 2.83	89.58
		AD	88.14 ± 2.09	74.17 ± 5.53	80.44 ± 3.55		

**Table 3.** AD Classification results of multi-modal learning using both audio and text. AD: Alzheimer’s Disease. Accuracy: mean and standard deviation of results of 5 rounds. Best: highest accuracy of all epochs in 5 rounds.

Model	Fusion / Training	Classes	Precision %	Recall %	Fl %	Accuracy *%*	Best %
Speech BERT	-	non-AD	66.13 ± 4.12	55.00 ± 4.86	59.94 ± 3.78	63.33 ± 3.12	79.17
		AD	61.48 ± 2.76	71.67 ± 4.86	66.12 ± 3.08		
BERT base	-	non-AD	78.46 ± 1.89	85.00 ± 2.04	81.60 ± 1.96	80.83 ± 2.04	85.42
		AD	83.64 ± 2.22	76.67 ± 2.04	80.00 ± 2.13		
Dual BERT	Add / Joint	non-AD	78.63 ± 1.77	85.83 ± 2.04	82.07 ± 1.79	81.25 ± 1.86	85.42
		AD	84.41 ± 2.13	76.67 ± 2.04	80.35 ± 1.95		
	Add / Separate	non-AD	78.96 ± 1.57	87.50 ± 2.64	82.99 ± 1.68	82.08 ± 1.66	85.42
		AD	86.05 ± 2.60	76.67 ± 2.04	81.06 ± 1.69		
	Concat / Separate	non-AD	80.39 ± 1.56	85.00 ± 3.33	82.57 ± 1.26	82.08 ± 1.02	85.42
		AD	84.21 ± 2.52	79.17 ± 2.63	81.54 ± 1.01		
	Concat / Joint (No pre-train speech)	non-AD	80.36 ± 2.06	85.00 ± 2.04	82.59 ± 1.56	82.08 ± 1.66	87.50
		AD	84.10 ± 1.91	79.17 ± 2.63	81.53 ± 1.83		
	Concat / Joint (Longformer)	non-AD	78.83 ± 4.18	88.33 ± 4.09	83.15 ± 1.79	82.08 ± 2.12	89.58
		AD	86.95 ± 3.38	75.83 ± 6.12	80.79 ± 2.74		
	Concat / Joint	non-AD	80.02 ± 1.16	86.67 ± 1.67	83.20 ± 1.01	82.50 ± 1.02	85.42
		AD	85.48 ± 1.46	78.34 ± 1.67	81.74 ± 1.10		
	Concat / Joint (BERT large)	non-AD	83.62 ± 4.25	82.50 ± 5.53	82.80 ± 1.76	82.92 ± 1.56	87.50
		AD	83.04 ± 3.97	83.33 ± 5.89	82.92 ± 1.86		
YAMNet + BERT base	Concat / Joint	non-AD	78.06 ± 2.53	85.83 ± 2.04	81.76 ± 2.22	80.83 ± 2.43	89.58
		AD	82.70 ± 3.65	82.50 ± 5.53	82.45 ± 3.07		

**Table 4.** Threshold-based strategy (20–30). The highest accuracy in training, the highest accuracy in testing, and the testing accuracy corresponding to the highest accuracy in training are in bold.

*T*	Accuracy (Training)	Accuracy (Testing) %
20	86.92	75.00
21	88.79	79.17
22	89.72	81.25
23	90.65	83.33
24	92.52	87.50
25	95.33	87.50
26	**97.20**	**89.58**
27	96.26	89.58
28	95.33	**91.67**
29	87.85	83.33
30	71.03	70.83

**Table 5.** Classification and regression results of multi-task transfer learning using CTP text. AD: Alzheimer’s Disease. Accuracy: mean and standard deviation of results of 5 rounds. Best: highest accuracy of all epochs in 5 rounds. RMSE: mean and standard deviation of Root-Mean-Square Errors of 5 rounds. Best RMSE: lowest RMSE of all epochs in 5 rounds.

Model	Pre-training	Settings	Accuracy %	Best %	RMSE	Best RMSE
Text (Luz et al, 2020)	-	Classification	75	-		
	-	Regression			5.20	-
BERT base	No	Classification	76.67 ± 1.56	81.25	-	-
		Regression	-	-	5.18 ± 0.04	4.65
		Multi-task	78.75 ± 1.56	83.33	4.70 ± 0.02	4.39
	Yes	Classification	80.83 ± 2.04	85.42	-	-
		Regression	-	-	4.15 ± 0.01	4.06
		Multi-task	80.83 ± 1.56	87.50	4.96 ± 0.01	4.20

**Table 6.** The best classification cases of the audio-based, text-based, and multi-modal models. AD: Alzheimer’s Disease. Accuracy: mean and standard deviation of results of 5 rounds. Best: highest accuracy of all epochs in 5 rounds.

Input	Model (with pre-training)	Classes	Precision %	Recall %	Fl%	Accuracy %	Best %
Audio	YAMNet	non-AD	69.60 ± 6.80	59.20 ± 7.73	63.40 ± 5.57	66.20 ± 4.79	83.33
		AD	64.40 ± 3.93	73.40 ± 8.82	68.60 ± 4.84		
Text	Longformer	non-AD	77.87 ± 3.75	90.00 ± 2.04	83.44 ± 2.33	82.08 ± 2.83	89.58
		AD	88.14 ± 2.09	74.17 ± 5.53	80.44 ± 3.55		
Audio + Text	Dual BERT Concat / Joint (BERT large)	non-AD	83.62 ± 4.25	82.50 ± 5.53	82.80 ± 1.76	82.92 ± 1.56	87.50
		AD	83.04 ± 3.97	83.33 ± 5.89	82.92 ± 1.86		

## 9 ACRONYM

AD: Alzheimer’s Disease
MCI: Mild Cognitive Impairment
CTP: Cookie Theft Picture
MFCC: Mel Frequency Cepstral Coefficient
ASR: Automatic Speech Recognition
MMSE: Mini-Mental State Examination
CNN: Convolutional Neural Network
GAP: Global Average Pooling
FC: Fully Connected
ADReSS: Alzheimer’s Dementia Recognition through Spontaneous Speech
NLP: Natural Language Processing
RMSE: Root-Mean-Square Error
IR: Image Recognition
GPU: Graphics Processing Unit
LSTM: Long Short-Term Memory
